# Carbon Nanostructure/Zeolite Y Composites as Supports for Monometallic and Bimetallic Hydrocracking Catalysts

**DOI:** 10.3390/nano12183246

**Published:** 2022-09-19

**Authors:** Roba Saab, Kyriaki Polychronopoulou, Dalaver H. Anjum, Nikolaos Charisiou, Maria A. Goula, Steven J. Hinder, Mark A. Baker, Andreas Schiffer

**Affiliations:** 1 Department of Mechanical Engineering, Khalifa University of Science and Technology, Abu Dhabi 127788, United Arab Emirates; 2 Center for Catalysis and Separations, Khalifa University of Science and Technology, Abu Dhabi 127788, United Arab Emirates; 3 Department of Physics, Khalifa University of Science and Technology, Abu Dhabi 127788, United Arab Emirates; 4 Department of Chemical Engineering, University of Western Macedonia, Koila, 50100 Kozani, Greece; 5 The Surface Analysis Laboratory, Faculty of Engineering and Physical Sciences, University of Surrey, Guildford GU2 4DL, UK

**Keywords:** carbon nanotubes, graphene, hydroprocessing, bi-functional catalyst, heptane

## Abstract

In this study, we examine the effect of integrating different carbon nanostructures (carbon nanotubes, CNTs, graphene nanoplatelets, GNPs) into Ni- and Ni-W-based bi-functional catalysts for hydrocracking of heptane performed at 400 °C. The effect of varying the SiO_2_/Al_2_O_3_ ratio of the zeolite Y support (between 5 and 30) on the heptane conversion is also studied. The results show that the activity, in terms of heptane conversion, followed the order CNT/Ni-ZY5 (92%) > GNP/Ni-ZY5 (89%) > CNT/Ni-W-ZY30 (86%) > GNP/Ni-W-ZY30 (85%) > CNT/Ni-ZY30 (84%) > GNP/Ni-ZY30 (83%). Thus, the CNT-based catalysts exhibited slightly higher heptane conversion as compared to the GNP-based ones. Furthermore, bimetallic (Ni-W) catalysts possessed higher BET surface areas (725 m^2^/g for CNT/Ni-W-ZY30 and 612 m^2^/g for CNT/Ni-ZY30) and exhibited enhanced hydrocracking activity as compared to the monometallic (Ni) catalyst with the same zeolite support and type of carbon structure. It was also shown that CNT-based catalysts possessed higher regeneration capability than their GNP-based counterparts due to the slightly higher thermal stability of the CVD-grown CNTs.

## 1. Introduction

Hydrocracking is a petrochemical process that converts undesirable heavy fuels into lighter value-added fuel products [1]. The process is facilitated through a bi-functional catalyst with acid and metal sites. Among various factors, the catalyst’s structure and composition are crucial for achieving high catalytic activity. The catalyst supports are typically micro- and mesoporous materials with a large surface area, such as amorphous silica/alumina and conventional/hierarchical zeolites [2].

Zeolites are often favored for hydrocracking reactions because of their relatively strong acidity, high thermal and hydrothermal stability, enhanced resistance to poisoning (e.g., by sulfur or nitrogen compounds found in crude oil), and reduced coke formation tendency [3]. Furthermore, the well-defined pore system of zeolites allows them to suppress or promote specific reactions depending on the size of the reactant molecules. Hierarchical zeolites, on the other hand, possess more than one level of pore size, including micro- and mesoporosity and, in some cases, even macroporosity [4]. When micro-porous zeolites are used as the catalyst support, the hydrocracking reaction may suffer from diffusion limitations, causing a reduction in the reaction rates [5]. Hence, considerable effort has been devoted to developing new types of zeolites with hierarchical pore systems that limit the diffusion path lengths and enhance the transport of large feed molecules to the active sites on the catalyst [6]. In particular, ultra-stable Y-type zeolites are widely used in hydrocracking reactions due to their high stability and activity at elevated temperatures [3].

The hydrocracking of heptane into lighter compounds is usually undertaken to utilize bi-functional catalysts [7,8,9]. For instance, a Ni-zeolite Y catalyst doped with graphene nanoplatelets (GNPs) was tested for hydrocracking of heptane [10], where the incorporation of GNPs into the catalyst elevated the conversion by 31% [10]. Further, a NiO-WO_3_/zeolite-Y catalyst was synthesized and tested for heptane hydrocracking in Ref. [9]. The authors showed that the use of a nano-sized zeolite Y support [11,12] (rather than conventional micro-sized zeolite) could increase the heptane conversion due to the relatively high BET surface area of the nano-sized crystals. Hierarchical micro-mesopore assemblies were reported in Ref. [13], where zeolite Y crystals with hierarchical porosity were dispersed on silicon carbide. The synthesized catalyst displayed increased rates of heptane cracking and olefin yields, along with slower deactivation, which was attributed to the hierarchical pore structure, allowing the faster diffusion and better accessibility of the reactants to the active sites. Further, NiMo/USY-Al_2_O_3_ was tested for hexadecane hydrocracking in Ref. [14], where the authors investigated correlations between ultra-stable zeolite Y (USY) recrystallization and catalytic performance. It is worth mentioning that Ni and/or Mo-based catalysts are extensively utilized as metal-promoting catalysts in both hydrocracking and hydrotreating processes [15,16,17,18,19,20] for a wide range of feeds.

Carbon deposition on the metal particles and into the support pores (i.e., coking) is one of the main reasons for the reduction in catalytic activity in hydrocracking reactions. The coking process initiates with the formation of a coke precursor on the metal phase of the catalyst. Following a sequence of reactions at the metal sites, extended carbon filaments (or filamentous coke) are formed, which can block some of the active metal sites, leading to a drop in the reaction rates [21]. Moreover, the deactivation of hydrocracking catalysts can also occur by the deposition of carbon on the acidic sites of the support, resulting in a reduction in the rate of cracking [21].

The integration of carbon nanotubes (CNTs) and other carbon nanostructures in hydrocracking catalysts has gained attention in recent years since it helps in generating mesoporosity in the zeolite support [22]. Furthermore, CNTs and graphene exhibit various unique properties, such as high thermal stability and conductivity [23,24], in addition to their high surface area [25]. A few approaches have been used for the synthesis of CNT/zeolite composites, such as growing zeolite crystals on existing CNTs [22,26,27] or growing CNTs on the surface of zeolites [28,29,30]. The latter method is well known and works by the catalytic decomposition of CH_4_, or other carbon-based feeds, on transition metals, such as nickel or cobalt [31]. Under favorable conditions, CH_4_ decomposes into atomic carbon and H_2_ gas following a sequence of surface reactions. Subsequently, the formed carbon atoms diffuse into the metal, leading to the growth of CNTs [31]. Moreover, a number of studies in the literature have merged Ni/NiO with graphene-like structures, observing improved performance in several applications attributed to the synergistic behavior between these material components. For instance, Ni and nitrogenated graphene were combined to obtain hybrid electrodes for hydrogen evolution reactions, which showed superior electro-catalytic activities as compared to neat Ni electrodes [32]. Functional groups present on the surface of graphene originate from the heteroatoms attached to the boundaries of graphene layers, which influence the electronic properties of the carbon material by providing electron acceptors or donors [33,34]. This potentially enhances π bonding, resulting in improved stability and electron transfer, which, in turn, improves the performance and durability of the catalyst [35,36]. Even though various preparation methodologies have been reported for CNT/zeolite composites [22,31,37,38] and for the integration of graphene into catalysts, the possible advantages of using them as supports in hydrocracking catalysts remain largely unknown.

Variations in the Si/Al ratios of the zeolite Y framework cause major changes in the zeolites’ protonation, which alters their acidity. It has been established that faujasite zeolites (including zeolite Y) with a lower Si/Al ratio have higher H^+^ selectivity and, thus, weaker acidity [39]. Although lowering the Si/Al ratio causes the number of acid sites in the zeolite to increase, each acid site becomes weaker as the cation sites are occupied by protons [40]. Moreover, it has been shown that not all the Brønsted acid sites contribute equally to the catalytic activity [41]. In particular, the presence of more isolated acid sites, as observed in zeolites with higher Si/Al ratios, has been associated with enhanced catalytic activity [42].

The effect of using bimetallic catalysts in hydrocracking reactions has been examined in the literature. Ni-W has been an attractive combination in various hydrogenation and hydro-denitrogenation reactions, for which favorable Ni:W ratios have been determined in previous studies [43]. The ideal Ni:W ratio is generally affected by the details of the metal-support interactions [44], but the optimal ratio for hydrocracking reactions has been determined to be within 0.25 and 0.36 in a number of studies [43,45].

Herein, we report the integration of CNTs (1D) and graphene nanoplatelets (GNPs) (2D) into monometallic (Ni) and bimetallic (Ni-W) zeolite-supported catalysts for heptane hydrocracking. The textural properties, structure, morphology, elemental composition, and chemical environment at the surface, as well as the thermal properties of the prepared catalysts, are determined, and their hydrocracking performance is studied in terms of heptane conversion, product selectivity, and time-on-stream stability. Given the importance of heptane hydrocracking as a model reaction, we aim to unveil the extent to which the reaction metrics (e.g., conversion, selectivity, and stability) are affected by the integration of carbon structures of different dimensionality (e.g., 1D CNTs, and 2D GNPs) into the catalytic system, and how these metrics are affected by the metal-support combination used in the reaction, which has not yet been reported in the literature, to the best of our knowledge. Another novel aspect of this work is the study of the regeneration capability of the catalysts, which is evaluated herein by performing three consecutive hydrocracking/regeneration cycles and measuring the heptane conversion and product selectivity percentages in each cycle. The study also aims to compare the reaction metrics of the CNT- and GNP-based catalysts to those of conventional metal–zeolite Y catalysts with equal nominal metal loading (in wt.%). The preparation techniques and methods adopted herein are selected based on their practicality and effectiveness in revealing the characteristics of the studied catalysts.

## 2. Experimental

### 2.1. Catalyst Synthesis

Two types of zeolite Y powder with SiO_2_/Al_2_O_3_ ratios of 5.1 (ZY5) and 30 (ZY30), respectively, were supplied by Zeolyst International (Conshohocken, PA, USA). Note that the aforementioned SiO_2_/Al_2_O_3_ ratios correspond to molar Si/Al ratios of 2.55 and 15, respectively. Graphene nanoplatelets (GNPs) of grade C-300 were supplied by Sigma Aldrich (St. Louis, MO, USA).

*Ni-ZY (5, 30) monometallic catalysts:* First, Ni was wet-impregnated on ZY5 and ZY30 individually, using nickel (II) nitrate hexahydrate, Ni(NO_3_)_2_.6H_2_O (Sigma Aldrich, St. Louis, MO, USA, >98.5%). The amount of Ni precursor was chosen to obtain 5 wt.% Ni on the zeolite support. Next, the two catalysts were dried for 12 h at 120 °C and then allowed to cool to room temperature. Calcination in air was then carried out on the dried catalysts at 500 °C for 5 h to obtain NiO-ZY. The obtained catalysts were then modified by growing CNTs or depositing GNPs, as explained below. Reference catalysts, such as NiO-ZY5 and NiO-ZY30, are reduced in pure H_2_ (50 mL/min) under atmospheric pressure and indicated in the manuscript as Ni-ZY5 and Ni-ZY30 for convenience.

*Ni-W-ZY(30) bimetallic catalyst:* Nickel (II) nitrate hexahydrate, Ni(NO_3_)_2_·6H_2_O (Sigma Aldrich, St. Louis, MO, USA, >98.5%), and ammonium metatungstate (AMT), (NH_4_)_6_[H_2_W_12_O_40_]·xH_2_O (Sigma Aldrich, St. Louis, MO, USA, >85%) were used as precursors for metal deposition via wet impregnation with the amounts of Ni and W precursors adjusted to obtain 5 wt.% metal loading (Ni:W in 1:3 weight ratio [44,45]). The synthesis was carried out only over the ZY30 support, based on the results obtained from our previous study [46]. The catalyst was later dried and calcined, as described above, forming NiO-WO_3_-ZY(30). The obtained catalyst was then modified by growing CNTs or depositing GNPs, as explained below. Reference catalyst, such as NiO-WO_3_-ZY30, is reduced in pure H_2_ (50 mL/min) under atmospheric pressure and indicated in the manuscript as Ni-W-ZY30 for convenience.

*CNT/Ni(W)-ZY (5,30) catalysts:* The as-synthesized NiO(-WO_3_)-ZY(5, 30) catalysts (without calcination) were placed in an Inconel fixed-bed reactor (PID Microactivity apparatus, Micromeritics, 9 mm internal diameter). The solid samples (≈0.5 g) were calcined in 20% O_2_/He at 500 °C for 1 h and then reduced in H_2_ at 500 °C for 45 min. Then the CNTs were grown in situ via CH_4_ decomposition (100% CH_4_ flow at 1 bar and 500 °C for 2 h). These catalysts will hereinafter be referred to as CNT/Ni-ZY5, CNT/Ni-ZY30, and CNT/Ni-W-ZY30, respectively. Further, the same synthesis protocol was repeated for the CNT/Ni-ZY30, using a different methane decomposition procedure, as follows: 100% CH_4_ flow at 500 °C for 4 h, 100% CH_4_ flow at 650 °C for 2 h, 100% CH_4_ flow at 650 °C for 4 h. The latter samples are referred to as CNT/Ni-ZY30 (500 °C, 4 h), CNT/Ni-ZY30 (650 °C, 2 h), and CNT/Ni-ZY30 (650 °C, 4 h), respectively.

*GNP/Ni(W)-ZY (5,30) catalyst synthesis:* Here, we followed a similar preparation method as described in [47]. Briefly, GNPs were dissolved in ethanol and kept under sonication for 1 h. Then, the NiO(-WO_3_)-ZY(5, 30) (as synthesized, after calcination) were added, separately, to the GNP solution in the weight ratio GNP to NiO(-WO_3_)-ZY of 1:15 and sonicated further for 3 h. The obtained solutions were later centrifuged at 6500 rpm for 10 min, and finally dried in an oven. The catalysts were reduced in situ preceding the catalytic testing in H_2_ at 350 °C for 3 h. In the following, these catalysts are referred to as GNP/Ni-ZY5, GNP/Ni-ZY30, and GNP/Ni-W-ZY30, respectively.

### 2.2. Catalyst Characterization

#### 2.2.1. Textural Properties

N_2_ adsorption–desorption isotherms at 77 K were obtained using a 3 Flex physisorption porosimeter (Micrometrics, Atlanta, GA, USA) to determine the Brunauer-Emmet-Teller (BET) surface areas, micropore volumes (via t-plots) and pore size distributions (via DFT method) of the prepared catalysts. All materials were degassed under vacuum at 120 °C for 5 h prior to the experiments. The t-plot statistical thickness method includes the evaluation of the N_2_ adsorbate layer thickness in terms of relative pressure, *P*/*P*_0_. The de Boer equation was then utilized to estimate the statistical thickness [48]:

(1) $$t\left(A\right)={\left[\frac{13.99}{0.034-\log\left(P/P_{0}\right)}\right]}^{1/2}.$$

Moreover, the average pore diameter was calculated by the ratio 4*V*_T_/*S*_BET_, in which *V*_T_ represents the specific total volume, whereas *S*_BET_ is the specific surface area [49].

#### 2.2.2. X-ray Diffraction (XRD)

Powder XRD analysis was employed to examine the structure of the crystalline catalysts using a Bruker D2 Phaser XRD apparatus (λ = 1.54184 Å, Cu-Kα), operated at 30 kV and 10 mA. The diffraction angle 2*θ* was varied between 5 to 80°.

#### 2.2.3. X-ray Photoelectron Spectroscopy (XPS)

XPS was used to study the oxidation states of the surface atoms and elemental composition of the catalyst surface. The analysis was performed on a Thermo-Fisher Scientific Instruments (East Grinstead, UK) K-Alpha^+^ spectrometer with a monochromatic Al Kα X-ray source (hν = 1486.6 eV). High-resolution core level spectra (obtained with an X-ray spot of 400 μm and a pass energy of 50 eV) were used to perform elemental quantification, as described in [42]. Note that all spectra were charge referenced against the C 1s peak at 285.0 eV.

#### 2.2.4. Raman Spectroscopy

Raman spectroscopy was performed to examine the characteristics of the integrated carbon structures (CNTs or GNPs) using a Raman-AFM combination (Witec Alpha 300 RA) operated at a laser wavelength of 532 nm.

#### 2.2.5. Scanning/Transmission Electron Microscopy (SEM/TEM)

High Resolution (HR) SEM was performed to examine the morphologies of the prepared catalysts using an FEI Nova NanoSEM 650. In order to gain further insight into the catalyst structure, HR-TEM images were obtained using an FEI Tecnai instrument (line resolution 0.20 nm). In addition, advanced TEM analyses were performed using a Titan 80-300 ST, operated at 300 kV and equipped with a spherical aberration (Cs) corrector for the image (CEOS CETCOR) and an energy filter (model GIF Quantum 963, Gatan, Inc., Pleasanton, CA, USA). X-ray energy dispersive (EDX) spectroscopy was performed to determine the elemental composition of the catalysts, while nanoscale elemental mapping was performed using electron energy loss spectroscopy (EELS) in scanning TEM (STEM) mode. The Microscopy Suite (GMS) software (version 3.2, Gatan, Inc., Pleasanton, CA, USA) was used for the acquisition and processing of the TEM data.

#### 2.2.6. Thermogravimetric Analysis (TGA)

TGA was performed on the fresh catalysts to examine their thermal properties using an SDT Q600 instrument under N_2_ flow of 20 mL/min. TGA was primarily employed to evaluate the thermal stability and decomposition kinetics of the materials through their weight loss with temperature.

### 2.3. Catalytic Testing

The hydrocracking experiments were performed in an Autoclave Engineers BTRS high-temperature and high-pressure testing system. The flow rates of the liquid and gas feeds were regulated using a high-pressure HPLC pump (Series I) and metering valves, respectively.

A continuous flow, fixed-bed tubular stainless-steel reactor was used to carry out the catalytic tests. Prior to the experiments, reduction in pure H_2_ (50 mL/min) under atmospheric pressure was performed in situ for the GNP-based catalysts, while the reduction of the CNT-based catalysts occurred during the synthesis process. The intended reaction temperature was set under a flow of high-purity N_2_ gas (100 mL/min), and then the system was pressurized with H_2_ gas at 5 bar. Liquid heptane (purity >99%) was allowed in the system at the rate of 0.035 mL min^−1^, together with a flow of pure H_2_ gas. A liquid–gas separator located downstream from the reactor was used to separate the liquid and gaseous reaction products.

The catalytic experiments were performed on fresh catalysts at a temperature of 400 °C, a H_2_ pressure of 5 bar, a Liquid Hourly Space Velocity (LHSV) of 4.2 h^−1^, and an H_2_:heptane molar ratio of 3:1. In each test, the catalyst was exposed to the reaction stream for 3 h, and the reaction products were examined at 1 h intervals. Note that the product analysis was performed as described in previous studies [10,42]. Moreover, stability tests were carried out over 20 h time-on-stream. Finally, some of the catalysts were tested for reusability by regenerating them in three cycles. In the first cycle, the catalysts were activated for 3 h under pure H_2_ flow, hydrocracked at 400 °C for 3 h, and then regenerated under 20% O_2_ at 450 °C for 2 h. The same steps were repeated for a total of three cycles, and the catalytic activity was measured in each cycle.

The heptane conversion was calculated as

(2) $$\%\;conversion=\left(\frac{X_{i}-X_{f}}{X_{i}}\right)*100$$

where *X_i_* and *X_f_* are the molar flow rates (mol/min) of heptane at the reactor inlet and outlet, respectively. The selectivity percentages, *S_i_*, of the main reaction products (methane, ethylene, ethane, propane, *iso*-butane, *n*-butane, and *n*-hexane) were calculated as follows:

(3) $$S_{i}\;\left(\%\right)=\left(\frac{C\;atoms\;in\;species\;i}{total\;C\;atoms\;found\;\;in\;the\;products}\right)*100$$

## 3. Results and Discussion

In the following Section 3.1, Section 3.2, Section 3.3 and Section 3.4, we first present the material characterization results and then discuss the effects of carbon structure integration, CNT growth conditions, Si/Al ratio in the zeolite support, and bimetallicity on the properties of the prepared catalysts separately. The same format will be followed in Section 3.5, where the catalytic performance is presented and discussed.

### 3.1. Textural Characterization

Table 1 summarizes the results obtained from N_2_ adsorption and desorption isotherms at 77 K, including the BET surface areas, micropore, mesopore, and total pore volumes of the prepared catalysts CNT/Ni-ZY5, GNP/Ni-ZY5, CNT/Ni-ZY30, GNP/Ni-ZY30, CNT/Ni-W-ZY30, and GNP/Ni-W-ZY30, respectively; the corresponding results for the neat zeolite support and the nanocarbon-free metal–zeolite catalysts are also included for reference. Note that the data for the CNT/Ni-ZY30 in Table 1 correspond to 500 °C/2 h CNT growth conditions. The textural characterization data for the other growth conditions (i.e., 500 °C/4 h, 650 °C/2 h, 650 °C/4 h) are presented in the supplementary Table S1. The experimental error in this measurement is ~0.075 cm^3^ g^−1^ for the specific volume and ~0.3 m^2^ g^−1^ for the specific surface area [50].

**Figure 1:**
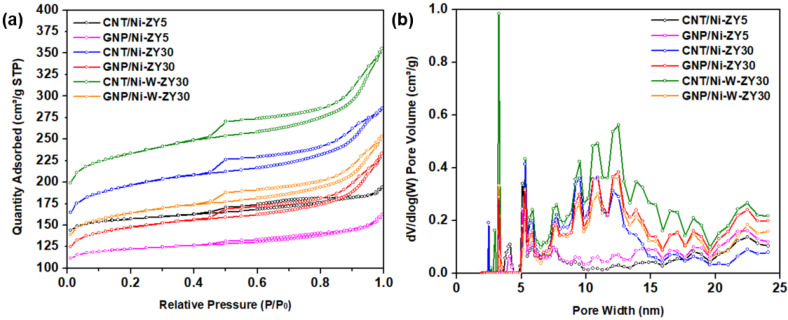
(**a**) N_2_ adsorption–desorption isotherms at 77 K, and (**b**) DFT pore size distributions (PSD) for the prepared catalytic materials.

Figure 1 presents the N_2_ adsorption–desorption isotherms (a), as well as the DFT pore size distribution (b) for the prepared catalysts. The isotherm plots for CNT/Ni-ZY30 prepared under different temperature/time conditions are shown in Figure S1. The isotherms for all the catalysts resemble type-IV isotherms, based on the IUPAC classification, portraying mesoporous materials, whereas the hysteresis loop that can be seen is of H3-type, attributed to the existence of slit-shaped pores. During desorption at ~0.45 relative pressure, a force is noticed on the hysteresis loop, revealed by the sharp slope, and this can be due to the tensile strength effect taking place for N_2_ at 77 K during this relative pressure range [51].

**Table 1:** Textural properties of the prepared catalysts.

Catalyst Composition	Ni (wt.%)	W (wt.%)	BET Surface Area (m^2^/g)	Pore Volume (cm^3^/g)		
				Micropore	Mesopore	Total
Reference supports/catalysts						
ZY5	-	-	399	0.21	0.01	0.22
ZY30	-	-	560	0.23	0.14	0.37
CNT	-	-	476 *	–	–	–
GNP	-	-	300 **	–	–	–
Ni-ZY5	5	-	409	0.19	0.06	0.25
Ni-ZY30	5	-	536	0.20	0.18	0.38
Ni-W-ZY30	1.67	3.33	608	0.23	0.21	0.44
CNT- and GNP-based catalysts						
CNT/Ni-ZY5	5	-	479	0.21	0.09	0.30
GNP/Ni-ZY5	5	-	372	0.16	0.09	0.25
CNT/Ni-ZY30	5	-	612	0.22	0.22	0.44
GNP/Ni-ZY30	5	-	458	0.17	0.19	0.36
CNT/Ni-W-ZY30	1.67	3.33	725	0.27	0.28	0.55
GNP/Ni-W-ZY30	1.67	3.33	511	0.18	0.21	0.39

* NanocylTM S.A. (Belgium), NC7000, industrial grade, C purity 90%, diameter 9.5 nm [52], ** Sigma Aldrich, graphene nanoplatelets, grade C-300, quality level MQ100 [10].

*Effects of carbon-structure integration (CNT* vs. *GNP):* As seen in Table 1, the CNT-based catalysts showed higher BET surface areas, as well as enhanced micropore, mesopore, and total pore volumes, as compared to the GNP-based catalysts with the same zeolite supports and metal loading. This can also be observed from the N_2_ adsorption–desorption isotherms (Figure 1a), where the CNT-based catalysts show higher N_2_ adsorption levels in comparison to their GNP-based counterparts, in consistency with their higher surface area and total pore volume. The data in Table 1 also show that the CNT-based composite catalysts had higher BET surface areas as well as larger micro-, meso- and total pore volumes as compared to their carbon-free zeolite-metal counterparts (e.g., CNT/Ni-ZY30 vs. Ni-ZY30), which can be attributed to the relatively high BET surface area of CVD-grown CNTs (~476 m^2^/g) [52]. The GNP-based catalysts, on the other hand, showed opposite trends (except for the mesoporosity, which slightly increased with GNP integration) since the GNPs have a significantly lower BET surface area (~300 m^2^/g according to the supplier’s datasheet) compared to the zeolite support (e.g., 560 m^2^/g for ZY-30). CNTs mainly grow on Ni particles and thus have a more directed growth which causes most of the pores in the zeolite to remain intact. On the other hand, GNPs were deposited on the surface of the zeolite, blocking some of its pores, as further discussed in Section 3.2. GNPs are composed of aggregates of platelets with particle diameters lower than 2 μm and macropores, a reason for which GNP-based catalysts show relatively higher average pore diameter (2.66, 3.11, and 3.04 nm) as compared to the CNT-based materials (2.48, 2.88, and 3.00 nm).

*Effect of CNT preparation conditions:* As seen in Table S1, varying the CNT growth conditions (500 °C/2 h, 500 °C/4 h, 650 °C/2 h, 650 °C/4 h) resulted in similar BET surface areas (ca. 576–612 m^2^ g^−1^) and almost equal porosities (total pore volume ca. 0.43–0.45 cm^3^ g^−1^); this is also reflected by the identical N_2_ adsorption–desorption isotherms shown in Figure S1. Although CNTs have a relatively smaller BET surface area than ZY30, the extra carbon grown on the catalyst surface seems to be negligible compared to the mass and volume of the ZY support, causing these insignificant differences.

*Effect of Si/Al ratio in zeolite support:* Increasing the SiO_2_/Al_2_O_3_ ratio of the zeolite Y support from 5 to 30 resulted in an increase in the BET surface area of the catalyst for the same metal loading and carbon deposition procedure followed (e.g., 479 m^2^ g^−1^ for CNT/Ni-ZY5 vs. 612 m^2^ g^−1^ for CNT/Ni-ZY30, see Table 1). The pore volume was also affected by the change in the Si/Al ratio; in particular, the mesopore volume significantly increased with the increase in the Si/Al ratio of the zeolites, which is anticipated to lead to changes in catalytic activity and product selectivity upon hydrocracking. These observations are consistent with previous work [53], where the authors report an increase in both surface area (from 666 to 797 m^2^ g^−1^) and mesopore area (from 120 to 171 m^2^ g^−1^) of faujasite (FAU) zeolites when increasing the Si/Al ratio from 6 to 30. Upon dealumination (increasing Si/Al), the FAU zeolite exhibits a modified framework, structure, and acidity, accompanied by an increase in its hydrophobicity, which affects the catalytic performance [53]. It is believed that the quantity of extra-framework Al species, created upon dealumination, plays a key role in enhancing catalytic activity. The N_2_ isotherms are between types I and IV for CNT/Ni-ZY5 and GNP/Ni-ZY5, while the isotherms of all other catalysts are of type IV. Since Type I is associated with microporous materials, these results suggest that the ZY5 supports can be considered as micro-porous, in accordance with their considerably lower mesopore volumes (0.09 cm^3^/g).

*Effect of monometallic (Ni)* vs. *bimetallic (Ni-W) promotion:* Further increases in the BET surface areas (Table 1), as well as higher N2 adsorption levels (Figure 1a), are reported for the bimetallic catalysts (CNT/Ni-W-ZY30 and GNP/Ni-W-ZY30) relative to their monometallic counterparts (CNT/Ni-ZY30 and GNP/Ni-ZY30). Moreover, the total pore volumes were found to be larger for bimetallic catalysts (0.39–0.55 cm^3^/g) as compared to the monometallic ones (0.36–0.44 cm^3^/g), where both the micropore and mesopore volumes are larger (Table 1). Surface area and porosity are known to be related to metal dispersion, which, in turn, strongly affects the catalytic activity of the supported mono/bimetallic catalysts [54]. The relatively large BET surface areas of the bimetallic catalysts are in agreement with the findings of previous studies [55] and are associated with enhanced metal dispersion. Note that the size and dispersion of the impregnated metals were examined using HR-TEM imaging, and these results will be discussed later in the following section.

### 3.2. Structure and Morphology Characterization

**Figure 2:**
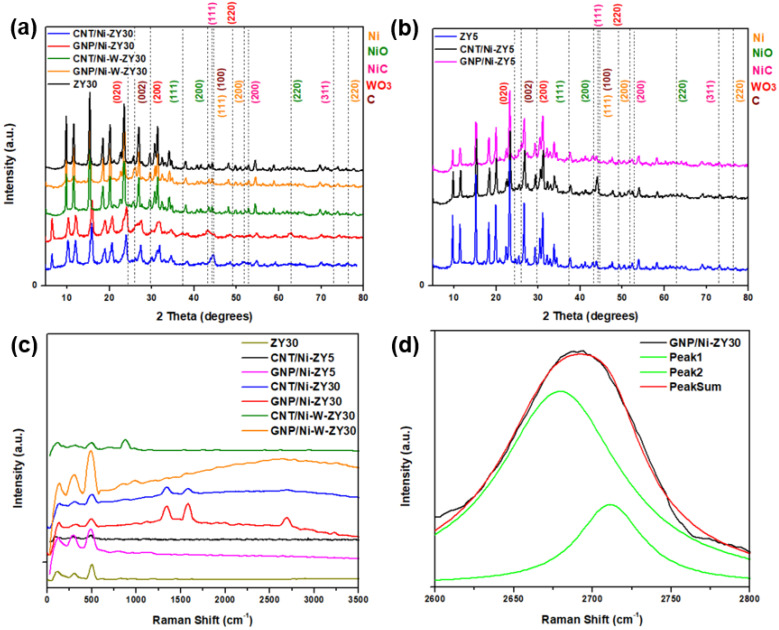
(**a**,**b**) XRD patterns and (**c**) Raman shifts in full range spectra recorded on the prepared solids, as well as (**d**) deconvolution analysis of the 2D Raman peak of the GNP/Ni-ZY30.

Figure 2a,b show the X-ray diffractograms for the prepared catalysts over a 2*θ* range of 5–80°. The major peaks associated with the zeolite framework are centered around 10.3°, 12.1°, 15.9°, 20.6°, and 24.1°, corresponding to the (220), (311), (331), (533), and (642) planes of zeolitic phase (ZY) [56]. These peaks do not seem to shift with the change in the Si/Al ratio of the zeolites.

Figure 2c presents the Raman spectra acquired for the prepared catalysts, while Figure 2d shows the deconvolution of the 2D Raman peak of the GNP/Ni-ZY30 catalyst. The low-shift bands located below 500 cm^−1^ in the composite catalysts are attributed to zeolite (Figure 2c). These bands are shifted towards higher wavenumbers (from 110 to ~125, 298 to ~300, and 495 to ~500 cm^−1^) as compared to the Raman of zeolite Y support before metal incorporation and carbon growth. This small shift originates from local lattice strains, leading to changes in local Al-O and Si-O bond strengths due to the presence of metal (Ni) and carbon structure [57].

**Figure 3:**
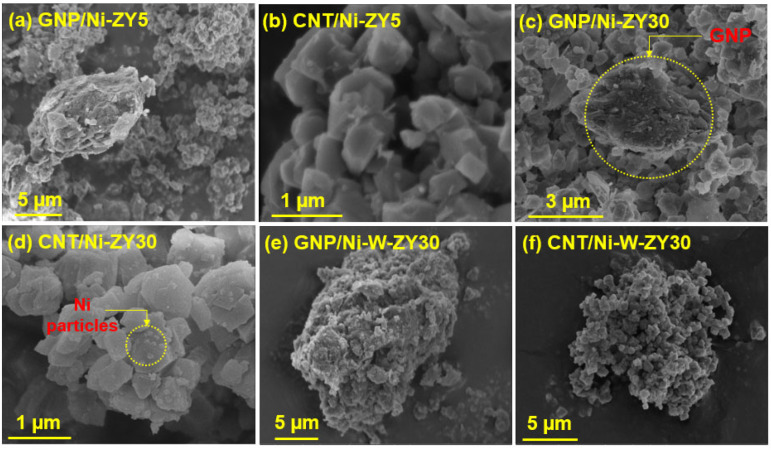
HR-SEM images (**a**–**f**) obtained for the prepared catalysts.

**Figure 4:**
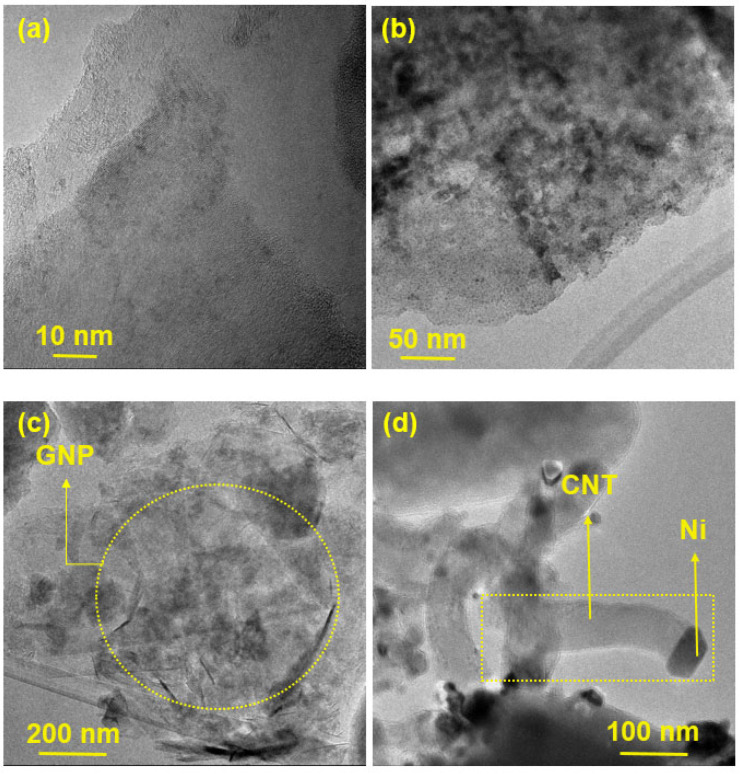
TEM images obtained on (**a**–**c**) GNP/Ni-W-ZY30 and (**d**) CNT/Ni-ZY30 catalysts.

Figure 3 presents HR-SEM images of the prepared catalysts, while TEM images of GNP/Ni-W-ZY30 and CNT/Ni-ZY30 are shown in Figure 4. The tetrahedral zeolite Y particles have identical morphologies, despite their varied Si/Al ratios, and can be clearly seen in the SEM images (Figure 3). However, the metals and metal-oxides (Ni, NiO; W, WO_3_) are barely detectible in the HR-SEM images due to their much smaller size, but they can be observed in the TEM images (Figure 4). Moreover, the TEM image of CNT/Ni-ZY30 (Figure 4d) reveals that the CNTs have an average diameter of ~50 nm, on which the Ni particle is located at the end of the tube. This suggests that the growth of CNTs lifted the metal particle upwards (i.e., away from the support) due to weak metal–substrate interactions [58]. GNPs, in turn, are short stacks of graphene sheets with platelet shapes, as shown in Figure 3a,c,e, and Figure 4c. The HR-SEM images reveal that the zeolite Y particles surround the GNP particles, which naturally have functional groups attached to their edges, such as ethers, carboxyls, or hydroxyls (as mentioned in the supplier’s datasheet).

**Figure 5:**
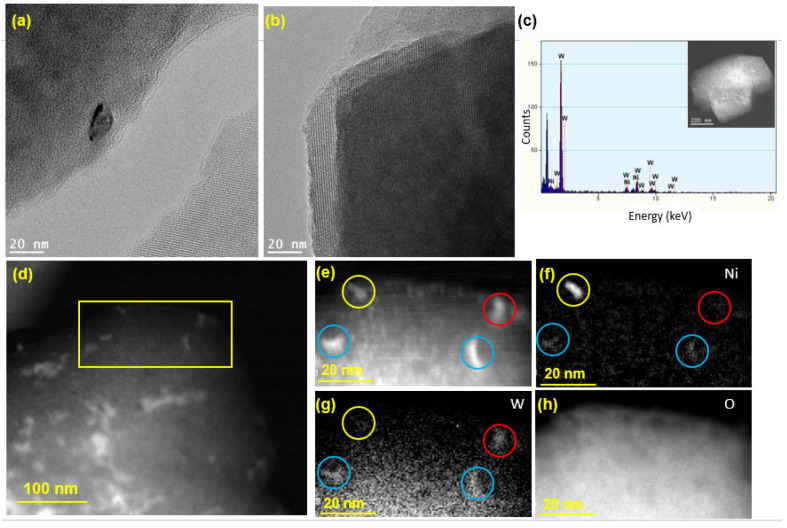
TEM analysis of Ni-W-ZY30: (**a**,**b**) HR-TEM images; (**c**) € EDX analysis; (**d**) low magnification HAADF STE€ (**e**) HAADF-STEM showing a selected area used for EELS mapping; (**f**–**h**) EELS elemental maps of Ni, W, and O.

The HR-TEM image in Figure 5a shows a Ni or W nanoparticle (size ~ 10 nm) loaded on the zeolite surface, while the crystalline structure of zeolite Y is clearly visible in Figure 5b, confirming that the crystallinity of the zeolite support was preserved following the synthesis of the catalyst. As seen from the TEM-EDX spectrum in Figure 5c, both Ni and W elements exist on the surface of the zeolite. To further examine the spatial distribution of Ni and W atoms on the zeolite, HAADF STEM imaging was performed, and the results are presented in Figure 5d–h. For the mapping of Ni and W elements, a smaller area was chosen, which is shown as a yellow box in Figure 5d. The image in Figure 5e shows four relatively large nanoparticles (marked by the colored circles), which were considered for EELS analysis to determine whether the impregnated Ni and W metals exist in a bimetallic form or as separate Ni and W phases. The EELS elemental mapping for the area exposes the presence of one pure Ni particle (yellow circle), one pure W particle (red circle), and two alloyed (bimetallic) Ni-W particles (blue circles). After repeating this analysis on different regions of the catalyst, the bimetallic Ni-W phase was estimated to account for 60% of the total metal loaded on the catalyst. Moreover, elemental mapping of O was carried out for imaging of the zeolite particle (Figure 5h). Table S2 (Supplementary Material) presents the EDX weight and atomic percentages for Ni-W-ZY30 determined after carrying out EDX-TEM over the region shown in Figure 5c. It can be seen from the table that the Si/Al ratios determined via EDX are in good agreement with their nominal values and that the W:Ni ratio (in wt.%) is around three, as expected.

*Effects of carbon-structure integration (CNT* vs. *GNP) and CNT preparation conditions:* The presence of sharp peaks in all the XRD patterns presented information that the crystallinity of the solids was preserved after the incorporation of GNP particles or the growth of CNTs (see Figure 2a,b). The NiO phase is characterized by three main diffraction peaks corresponding to the (111), (200), and (220) planes, while the Ni phase is characterized by diffraction peaks corresponding to the (111), (200), and (220) planes but at different 2*θ* values, which are presented in Figure 2a,b. Throughout the CNTs growth process, we expect the formation of a separate Ni_x_C phase, and this is confirmed by the powder XRD results. Diffraction peaks attributed to the presence of the Ni_x_C_y_ phase are shown in Figure 2a,b. Based on the stoichiometry y/x, various XRD patterns can be observed for Ni_x_C_y_. Particularly for the NiC phase (y/x = 1), the major diffraction peaks correspond to the (111), (200) and (311) planes [59]. The NiC crystal phase diffraction peaks can be seen in the CNT- and GNP-based composites, particularly the (111) and (200) planes; yet, since these peaks are very close to those associated with Ni and CNTs, the existence of the NiC phase will be further discussed along with the XPS results (Section 3.3). GNPs are indexed by a major peak at 2*θ* of 26° corresponding to the (002) plane, comparable to the peak reported in the literature for GNP [60]. In contrast, CNTs are characterized by two major diffraction peaks at 2*θ* values of 26.0 and 43.4°, corresponding to (002) and (100) planes [61], respectively. Since these peaks are close to those associated with zeolite Y and Ni, alternative characterization techniques (Raman spectroscopy, TEM) will be used to confirm the existence of CNTs on the catalysts. The XRD patterns for CNT/Ni-ZY30 prepared at different CH_4_ decomposition conditions (500 °C/2 h, 500 °C/4 h, 650 °C/2 h, 650 °C/4 h) are presented in Figure S2 (Supplementary Material), showing nearly identical diffraction peaks with similar peak intensities.

Carbon-based materials often show characteristic bands in the Raman spectra. While the G-band is associated with the graphitic sp^2^ bonded carbon, the D-band provides information on the presence of defective sp^3^ bonded carbon. Note that the defects present in the CNTs and GNPs are vital for improving the catalytic activity of metal-supported catalysts, which is attributed to the strong interaction created between the metal nanoparticles and the defective carbon-based surfaces [62,63]. As seen from Figure 2c, the G-band, D-band, and G’ band for the CNT/Ni-ZY30 occur at ~1577, 1345, and 2689 cm^−1^, respectively. The intensity ratio I_D_:I_G_ for CNT/Ni-ZY30 is ~1.3, which is consistent with typical values for CVD-grown CNTs (0.5–1.3) [64]. The Raman shifts of GNP/Ni-ZY30 show that the G, D, and G’ bands occur at around 1580 cm^−1^, 1338 cm^−1^, and 2682 cm^−1^, respectively, similar to the values found in the literature for GNP [65]. The I_D_:I_G_ ratio is around 0.67 for GNP/Ni-ZY30 (and 0.6 for the neat GNP), indicating a minor increase in defects upon catalyst preparation, presumably caused by the destruction of the 2D structure upon sonication. Further, all bands exhibit substantial broadening, which is typically associated with a disorder in the structure and/or strong surface modification [66]. CNTs grown on the ZY5 support and on the bimetallic Ni-W-ZY30 do not possess clearly visible G and D bands, suggesting a relatively small quantity of grown CNTs in comparison to the volume of the zeolite Y in these catalysts. However, this shall be further confirmed and discussed in the XPS analysis (Section 3.3). Figure 2d shows the deconvolution of the 2D Raman peak of the GNP/Ni-ZY30, noting that the shape of this peak and its intensity relative to the G-band are related to the layer thickness of graphene [67]. The shape of the peak and the fitting by two Lorentzian peaks reveal the bulk graphitic nature of GNPs, which indicates that, although sonicating GNPs upon preparation may have exfoliated some of the GNPs, this exfoliation method is not sufficient for lowering the number of graphene layers to below 10.

*Effect of Monometallic (Ni)* vs. *Bimetallic (Ni-W) Promotion:* Cubic NiO phase is characterized by diffraction peaks at 37.5, 42.8, and 62.8° 2*θ*, which correspond to (111), (200), and (220) planes, whereas hexagonal WO_3_ phase is indexed as (020), (200), and (220) occurring at 23.7, 29.7, and 49.3° 2*θ* [68] (see Figure 2a,b). NiO and Ni phases can be observed in the monometallic CNT/Ni-ZY30 and GNP/Ni-ZY30 (Figure 2a), whereas the NiO, Ni, WO_3_, and W phases are expected to exist in the bimetallic CNT/Ni-W-ZY30 and GNP/Ni-W-ZY30. Yet, since the zeolite structure possesses high crystallinity, the peak intensities representing the metallic phases are relatively low, which makes it challenging to confirm their existence using only the XRD technique. Moreover, NiO/Ni phases are hardly visible in the bimetallic catalysts, which can be attributed to the presence of Ni in relatively small quantities in comparison to the monometallic catalysts. Although the presence of a bimetallic Ni-W phase was shown via HAADF STEM (Figure 5d–h), the diffraction peaks corresponding to this phase were not visible in the XRD patterns, which can be attributed to the fact that the XRD analysis was performed on the calcined, non-reduced catalysts, whereas TEM was carried out on calcined and reduced samples. Hence, XPS analysis will be employed (in Section 3.3) to examine the surface elemental composition of the catalysts.

The Raman spectra of the monometallic catalysts show a higher intensity and more evident peaks for the G-band, D-band, and G’ band as compared to their bimetallic counterparts (see Figure 2c). This suggests that the growth of CNTs and doping of GNPs was less effective over bimetallic catalysts. CNT growth by CH_4_ decomposition is expected to take place more effectively on Ni rather than W nanoparticles, which explains their inhibited growth in the bimetallic catalysts having a relatively smaller quantity of Ni with respect to the monometallic ones. Transition metals such as Ni, Fe, and Co are extensively used for CH_4_ decomposition because their partially filled 3d orbitals can accept electrons from the C-H bond of CH_4_ [69]. Yet, most of the work in the literature concentrated on Ni-based catalysts owing to their high catalytic activity and capability of producing CNTs.

### 3.3. XPS Analysis

**Figure 6:**
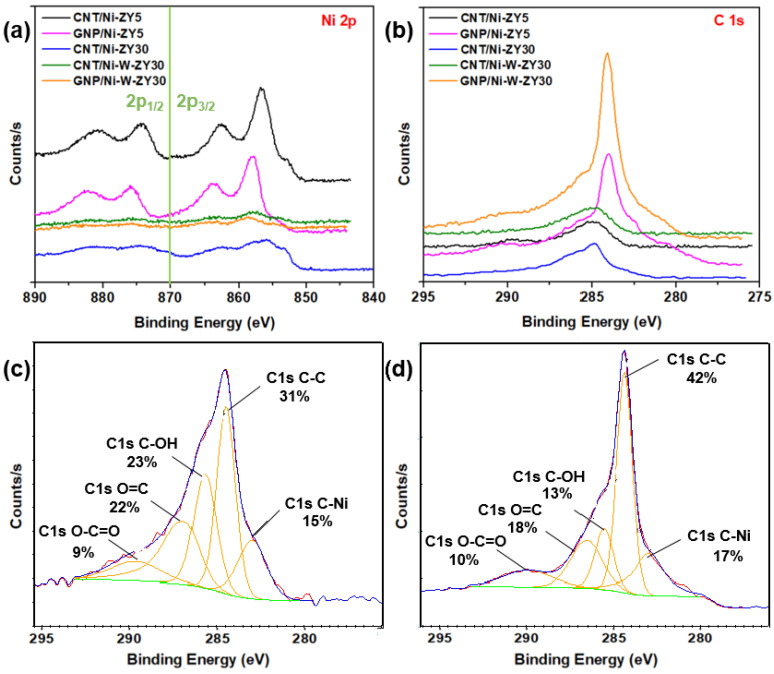
XPS core level spectra: (**a**) Ni 2p and (**b**) C 1s for the prepared catalysts, including peak fits showing the presence of the sp^2^ hybridized carbon in (**c**) CNT/Ni-ZY30 and in (**d**) GNP/Ni-ZY5.

The chemical environment and elemental composition of the catalyst surface were investigated using XPS analysis. XPS core level spectra of Ni 2p and C 1s are shown in Figure 6a,b, while Figure 6c,d show C 1s peak fits for CNT/Ni-ZY30 and GNP/Ni-ZY5, respectively. The binding energies and atomic percentages for each catalyst are presented in Table 2 for comparison. The Ni 2p_3/2_ peak is detected at 855–858 eV for all catalysts. Various nickel oxide/hydroxide species exist, possessing similar binding energies and complex peak shapes [70,71,72]. The characteristics of the Ni 2p_3/2_ peaks for all catalysts imply the existence of mainly Ni^2+^ oxides/hydroxides at the surface of the solids, noting that the hydroxides can be formed as a layer on the metal oxide surface due to ambient air exposure.

*Effect of carbon structure integration (CNT* vs. *GNP):* The Ni 2p spectra reveal that a low binding energy shoulder (~853.0 eV) exists for CNT-based composites attributed to Ni^0^, and this is less evident for GNP-based catalysts (see Figure 6a). As the CNTs are grown on the catalyst surface, the hydrogen gas formed during CH_4_ decomposition may further reduce the NiO phase that was not previously reduced, converting some Ni^2+^ species into Ni^0^, and leading to the more evident Ni^0^ peak. The surface carbon concentrations for CNT-based catalysts are between 2.5–5.8 at.%, arising from the integration of CNTs into the catalyst structure, whereas those for GNP-based catalysts are between 10.2–11.3 at.%, as seen from Table 2. When comparing the carbon-based catalysts to the reference ones, it can be observed that the methane composition was only reasonably effective over the Ni-ZY30, where the increase in surface carbon is significant, in line with the results obtained from the Raman spectra. From the C 1s spectra (Figure 6b) and corresponding peak fits (Figure 6c,d), we observed a strong peak for the GNP- and CNT-based catalysts at binding energies of 284 and 285 eV, respectively, along with a smaller peak at lower binding energy, ~283.6 eV. While the former peaks correspond to the CNT and GNP structures [73], the latter are mainly attributed to Ni carbides [74] (and probably W carbide in the bimetallic composites, as both carbides have similar binding energies). Moreover, other peaks exist at higher binding energies, and these are related to oxidized carbon species (see Figure 6c,d). The area percentages under the fitted peaks for CNT/Ni-ZY30 and GNP/Ni-ZY5 are as follows: 31% and 42% for the C-C peak, 23% and 13% for the C-OH peak, 9% and 10% for the O=C-O peak, 22% and 18% for O=C, and 15% and 17% for C-Ni, respectively. We also note that the Si and Al surface concentrations are lower for the GNP-based catalysts as compared to their CNT-based counterparts due to the bulky GNPs partly covering the catalyst surface and thus making it difficult to analyze the surface chemistry of the zeolite.

*Effect of Si/Al ratio in zeolite support:* The Si 2p, Al 2p, and O 1s peaks (not shown in Figure 6) exist at binding energies of around 103.6 eV, 74.8 eV, and 532.9 eV, respectively (see Table 2), in accordance with those for sodalite, (Al_0.2_Si_0.8_)O_2.2_, an aluminosilicate of a similar composition [75]. The Si/Al atomic ratios of the zeolites, as obtained from our XPS analysis (Table 2), differed from their nominal ratios since XPS measures the surface elemental compositions of the catalyst, which can deviate from the average values of the bulk. Carbon deposition, and thus CNT growth on zeolites, occurs primarily over the strong acid site density species (Si–OH–Al) [76,77,78]. This, in turn, indicates a favored growth of CNTs on the Al-rich species, leading to a higher effective surface Si/Al ratio of the CNT-based catalysts compared to their nominal Si/Al ratios, as affirmed by our XPS results for CNT/Ni-ZY30 and CNT/Ni-W-ZY30 (Table 2). Furthermore, this explains why CNT growth was more pronounced over the higher Si/Al ratio support, Ni-ZY30 (5.8 at.%, Table 2), rather than Ni-ZY5 (2.5 at.%, Table 2), since the former has stronger acid sites (even though their quantity is less). Note that an increase in the surface Si/Al ratio is associated with a decrease in surface acidity and a concomitant increase in the strength of the acid sites [79]. The Si/Al ratio in ZY5 was found to be identical for both monometallic and bimetallic catalysts (1.1) but varied between 16.8 and 25.4 for the ZY30 supported catalysts (Table 2).

*Effect of monometallic (Ni)* vs. *bimetallic (Ni-W) promotion:* When analyzing the monometallic and bimetallic catalysts with the same zeolite support (i.e., CNT/Ni-ZY30 vs. CNT/Ni-W-ZY30), it is observed that the latter experiences a shift in the Ni 2p_3/2_ peak from 856.8 to 857.8 eV, indicating the higher presence of Ni^3+^ [80]. Furthermore, the shoulder at ~853.0 eV with low binding energy arising from Ni^0^ is more pronounced in the monometallic catalyst, CNT/Ni-ZY30, despite the fact that all the Ni 2p spectra peaks associated with the bimetallic catalysts are barely observed since Ni exists in relatively small intensities. In bimetallic catalysts prepared herein, the Ni concentrations are reduced by the introduction of W metal to the catalyst, leading to lower amounts of Ni residing on the surface. The W 4f peak decomposition was performed using two equal-width peaks with the binding energy of W 4f_7/2_ centered at 36.3 eV. The spin–orbit splitting of the doublet is 1.96 eV, with a peak ratio of 4:3. An oxidation state of +6 of the W atoms gave rise to the position and shape of these W 4f peaks associated with the WO_3_ chemical state [81].

### 3.4. Thermal Analysis (TGA)

**Figure 7:**
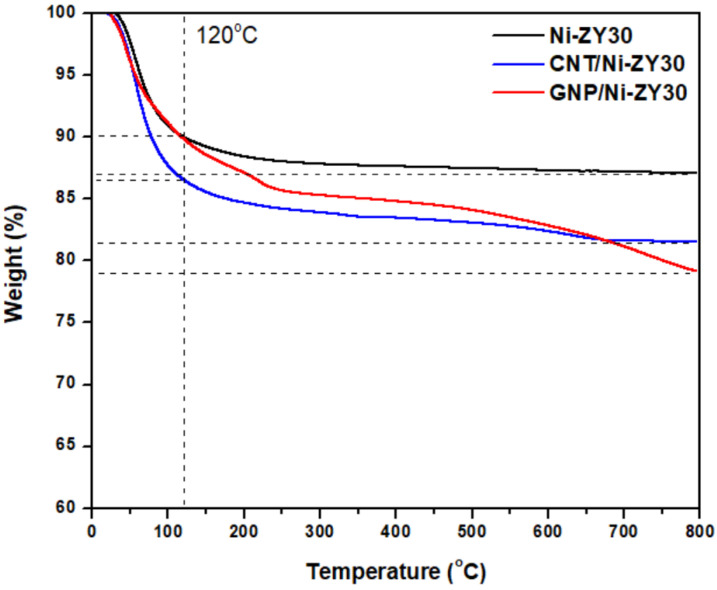
TGA profiles of Ni-ZY30, CNT/Ni-ZY30, and GNP/Ni-ZY30.

TGA was performed on CNT/Ni-ZY30, GNP/Ni-ZY30, and Ni-ZY30 to assess the thermal stability of the carbon-based catalysts and compare them to those observed for the conventional metal promoted zeolite Y support, Ni-ZY30. As seen from the TGA curves in Figure 7, the materials exhibit two regions of weight loss, one occurring at temperatures <120 °C and the other one between 120–800 °C. While Ni-ZY30 and GNP/Ni-ZY30 both exhibit a weight loss of 10% in the first region (<120 °C), CNT/Ni-ZY30 experiences a slightly larger loss of 13.5%. This weight loss can be attributed to the evaporation of adsorbed water in all the materials, and as the CNT/Ni-ZY30 has a relatively higher pore volume (see Table 1), it also shows a slightly larger loss of water vapor. In the second region (120–800 °C), Ni-ZY30, CNT/Ni-ZY30, and GNP/Ni-ZY30 show weight losses of 3, 5, and 11%, respectively. These drops are attributed to slight zeolite decomposition, as revealed by the 3% weight drop of Ni-ZY30, as well as carbon decomposition in the other catalysts, in accordance with the patterns reported in the literature for similar materials [82,83,84]. It is also seen from Figure 7 that the weight loss in CNT/Ni-ZY30 and GNP/Ni-ZY30 progresses more rapidly, starting at ~500 °C and ~450 °C, respectively, possibly due to the thermal degradation of the carbon structures present on the catalyst surface. The latter decomposition is more pronounced for the GNP/Ni-ZY30 due to the relatively large amount of carbon present on the surface of the GNP-based catalysts, as revealed by our XPS analysis (see Table 2).

### 3.5. Catalytic Performance

**Figure 8:**
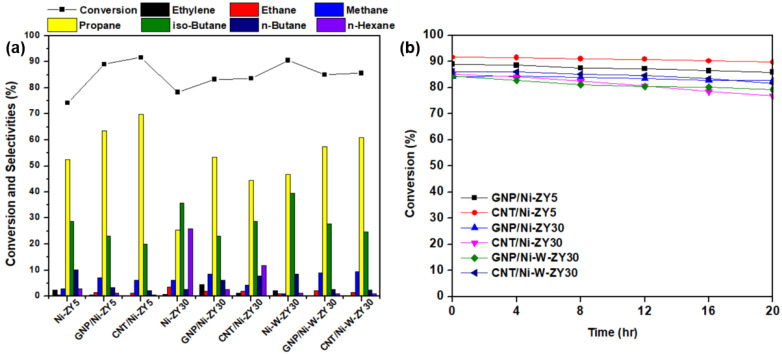
(**a**) Conversion and selectivity percentages of the main products for the prepared catalysts and the reference catalysts, and (**b**) heptane conversion over 20 h time-on-stream.

Figure 8 presents the heptane conversion and selectivity percentages of various reaction products for the catalysts tested at 400 °C, in addition to the stability results performed for 20 h time-on-stream. The heptane conversion ranges between 83–92% for the six tested carbon-based catalysts, with CNT/Ni-ZY5 having the highest activity and GNP/Ni-ZY30 the lowest. The data obtained for the reference catalysts without nanocarbon incorporation, namely Ni-ZY5, Ni-ZY30, and Ni-W-ZY30, are added to Figure 8 as well. The stability over time-on-stream is fairly identical for the tested catalysts, as they all show high stability even after 20 h of heptane hydrocracking, where the percentage drop in conversion is between 2–9% for the various catalysts. This drop is mainly caused by the deposition of coke on the catalyst surface and inside the pores, decreasing the concentration of active sites, either via Ni carburization or porosity plugging. Note that the reactivity and product selectivity of a typical bi-functional hydrocracking catalyst highly relies on the balance between the metal and acid functions [85]. This is because reactants go through successive reactions, for which the mechanism is greatly affected by the balance between metal and acid functions. For instance, very low acidity with respect to the metal function mostly results in hydrogenolysis, such as cracking on the metal sites followed by hydrogenation; in contrast, very strong acidity gives rise to several acid-catalyzed reaction steps in a single catalytic cycle [85]. It should also be mentioned that the lack of symmetric carbon distributions in the products (e.g., unequal amounts of C_1_ and C_6_, or C_2_ and C_5_, etc.) indicates the occurrence of secondary cracking during the experiments. The reproducibility of the catalytic results for the tested catalysts was assessed by repeating the experiments at least three times on fresh catalysts, and it was found that individual measurements lie well within the 95% confidence intervals calculated for the mean value.

**Figure 9:**
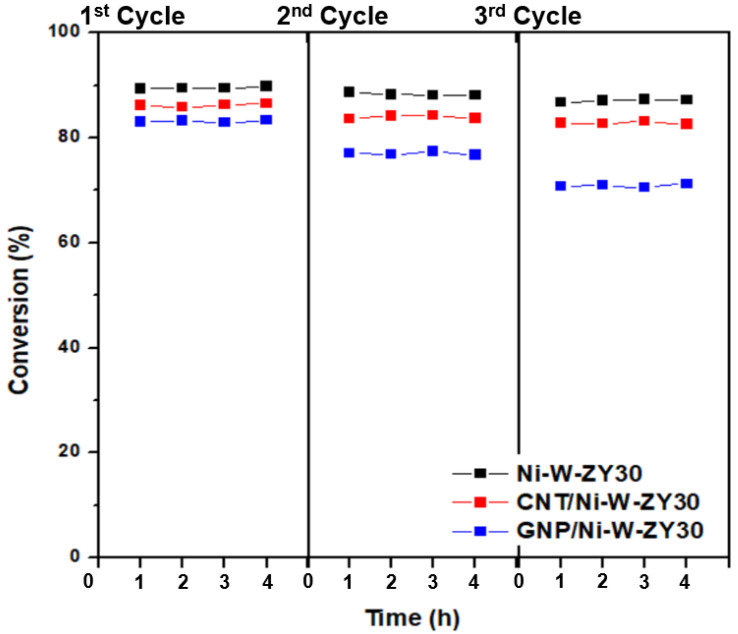
Conversion (%) over Ni-W-ZY30, CNT/Ni-W-ZY30, and GNP/Ni-W-ZY30 catalysts, after regenerating them in three repeated cycles.

An experimental procedure was also devised to evaluate the regeneration potential of the carbon-based catalysts, and the results are shown in Figure 9. It is observed that, after regenerating the catalysts for three cycles, the heptane conversion dropped in different amounts for Ni-W-ZY30, CNT/Ni-W-ZY30, and GNP/Ni-W-ZY30 catalysts, and this will be further discussed below.

*Effects of carbon-type (CNT* vs. *GNP) and CNT preparation conditions:* The activity results reveal that for the same supports and metal loadings, carbon-based catalysts show enhanced catalytic activity and heptane conversion as compared to the reference catalysts, particularly for the monometallic catalysts, Ni-ZY5 and Ni-ZY30, suggesting the role that nanocarbon plays in the composite catalyst as a coke reservoir, which was revealed in a previous study [46]. In turn, CNT-based catalysts show slightly improved heptane conversion over GNP-based ones for all the tested materials. This improvement is most pronounced among GNP/Ni-ZY5 (89%) and CNT/Ni-ZY5 (92%) combinations. This can be attributed to the higher BET surface area and pore volumes (micro-, meso-, and total volumes) of the CNT-based catalysts (see Table 1). These variations in textural properties of the CNT-modified catalysts can also result from the different preparation routes employed for each catalyst. Particularly, CNT-based catalysts were treated at a substantially higher temperature range (at the CH_4_ decomposition stage) as compared to GNP-based ones. The difference in thermal treatment history of the samples might cause enhanced zeolite-Y transformations in the case of CNT-modified systems, such as residual decomposition and partial structure collapse. In turn, substantial differences in nanocarbon/precatalyst weight ratio might arise, making the evaluation of the CNT and GNP effect rather challenging. Furthermore, a lower rate of coke formation is expected for the CNT-based catalysts, as the CNT structure acts as a coke reservoir for the produced carbonaceous species upon hydrocracking, thus preventing some of these species from blocking the active sites and consequently improving the activity of the catalyst. In addition, the accessibility of the acid sites is expected to be higher in the CNT-based catalysts compared to the ones doped with GNPs, mainly because of the way the carbon nanostructure is grown on the existing Ni/ZY catalyst. The GNPs deposited on the catalyst surface may block some of the acid sites on the zeolite, resulting in lower cracking rates. As the hydrocracking reaction proceeds, some of the Ni particles are slowly released through decomposition of the Ni_x_C phase into Ni and C (which is more pronounced in CNT-based catalysts as discussed in Section 3.3), thus protecting a portion of the active metal sites from deactivation [46]. It should also be noted that variations in the pore structure of the catalysts generally result in different product distributions [86]. GNP-based materials reveal higher selectivity towards ethane, ethylene, and methane as compared to CNT-based catalysts for identical metal loading and support.

Varying the preparation conditions of the CNT/Ni-ZY30 catalyst (CH_4_ decomposition: 500 °C/2 h, 500 °C/4 h, 650 °C/2 h, 650 °C/4 h) seems to have very little effect on the catalytic performance during the heptane hydrocracking experiments, as seen from the supplementary Figure S3. The heptane conversion levels of the CNT/Ni-ZY30 catalysts prepared under different CNT-growth conditions lie between a narrow band of 83–84%, with minor differences in selectivity percentages. For example, as the CH4 decomposition temperature and duration increase (from 500 to 600 °C and from 2 h to 4 h), the selectivity towards propane and n-hexane decreases while that towards iso-butane increases.

Upon regeneration, the oxidation of the coke deposits (formed during the hydrocracking reaction) is likely not complete, causing reduced activity in the following hydrocracking cycle, and this was also observed for the catalysts tested herein (see Figure 9). The Ni-W-ZY30 showed the lowest conversion drop over the three cycles (89.4% to 88.7% to 86.7%), followed by CNT/Ni-W-ZY30 (86.2% to 83.6% to 82.8%), and finally, GNP/Ni-W-ZY30 showed the highest drop in conversion (83.0% to 77.1% to 70.8%), and thus the lowest regeneration capability. The latter can be attributed to the lower thermal stability of the GNPs. The corresponding selectivity percentages are reported in the supplementary Figure S4, showing only minor variations over the three cycles for the Ni-W-ZY30, CNT/Ni-W-ZY30, and GNP/Ni-W-ZY30 catalysts.

*Effect of Si/Al ratio in zeolite support:* When comparing the Si/Al ratio of the zeolite Y supports for the same metal loading and type of carbon deposited (GNP or CNT), it can be observed that the ZY5-supported catalysts generally show superior performance over the ZY30 materials (see Figure 8). For instance, the conversion of heptane over the GNP/Ni-ZY5 is 89%, whereas it stands at 83% for the GNP/Ni-ZY30. Similarly, while the heptane conversion on the CNT/Ni-ZY5 is 92%, it stands at only 84% for the CNT/Ni-ZY30. Although the BET surface areas and pore volumes of the ZY30-based catalysts (higher Si/Al) are larger than those for ZY5 (see Table 1), the latter supports induce higher acidity, which is crucial during hydrocracking. As mentioned above, by increasing the surface Si/Al ratio of a zeolite, its surface acidity decreases along with an increase in the strength of these acid sites [79]. Since CNTs have a preferential growth over the strong Brønsted acid sites, which are more abundant in the ZY30, as compared to ZY5, as discussed in Section 3.3, a larger number of the weaker acidic sites are still available in ZY5 after growing CNTs for hydrocracking, which outweighs the lower quantity of stronger acid sites available in ZY30-based catalysts, causing the former’s enhanced catalytic activity. This might not be the case, though, when comparing zeolite catalysts of different Si/Al ratios that lack CNTs/GNPs, since the higher strength of fewer acid sites might overcome the higher quantity of weaker sites, as reported in [41]. Hence, upon the growth of CNTs over the lowest Si/Al ratio support (ZY5), the catalytic activity of the obtained catalyst was the highest, in which CNTs act as coke reservoirs that tend to reduce the coke deposition on the active catalyst surface. As for the selectivity toward various products, higher Si/Al ratio supports (e.g., ZY30) are more selective towards ethylene, ethane, n-butane, and n-hexane but less selective towards propane (see Figure 8b).

*Effect of monometallic (Ni)* vs. *bimetallic (Ni-W) promotion:* The effect of monometallic vs. bimetallic catalysts can be examined by comparing the performance of GNP/Ni-ZY30 to GNP/Ni-W-ZY30, or alternatively, CNT/Ni-ZY30 to CNT/Ni-W-ZY30. In both cases, the bimetallic system catalysts show enhanced hydrocracking activity to some extent: 83 vs. 85% and 84 vs. 86%, respectively (see Figure 8a). The improved performance is not only due to the higher BET surface areas and pore volumes of the bimetallic catalysts (see Table 1) but also because of the superior effect of the Ni-W mixed phase in an appropriate ratio, as widely reported in the literature [43,44]. The selectivity percentages of the products for the monometallic materials compared to the bimetallic with the same type of carbon deposited and zeolite support show that the monometallic catalysts are more selective towards ethylene, n-butane, and n-hexane but less selective towards methane and propane.

## 4. Conclusions

Zeolite-supported bi-functional catalysts with integrated carbon nanostructures (CVD-grown CNTs or deposited GNPs) were synthesized and tested for hydrocracking of heptane at a temperature of 400 °C. A series of catalysts were prepared, varying the type of loaded metals (monometallic Ni and bimetallic Ni-W) and altering the SiO_2_/Al_2_O_3_ ratio of the zeolite Y support (5 and 30). The bulk and surface properties of the prepared materials were characterized and used to study the sensitivity of the reaction metrics on the presence of carbon nanostructures, varied metal composition, and the SiO_2_/Al_2_O_3_ ratio in the zeolite Y support.

The heptane conversion was found to vary between 83–92% for the tested materials and was highest for CNT/Ni-ZY5 and lowest for GNP/Ni-ZY30. Further, the CNT-based catalysts exhibited a slightly higher heptane conversion than their GNP-based counterparts, which was attributed to the higher BET surface area and pore volumes of the CNT-based catalysts. Varying the parameters of the CVD growth process had a negligible effect on the catalytic performance of the CNT-based catalysts. It was also found that the zeolite-supported catalysts with lower SiO_2_/Al_2_O_3_ ratio (ZY5) had a superior catalytic performance as compared to the higher SiO_2_/Al_2_O_3_ ratio support (ZY30) materials, which was attributed to the higher surface acidity of ZY5. Additionally, bimetallic catalysts exhibited enhanced hydrocracking activity, to some extent, as compared to the monometallic ones, which is ascribed to the higher BET surface areas reported for the bimetallic catalysts.

Upon regeneration of the catalysts, the heptane conversion of Ni-W-ZY30 fell from 89% to 87% after the three repeated hydrocracking/regeneration cycles, while the drop for the CNT/Ni-W-ZY30 was somewhat more pronounced (from 86% to 83%). The lowest regeneration capability was reported for GNP/Ni-W-ZY30, where the heptane conversion dropped from 83% to 71% due to the slightly lower thermal stability of the deposited GNPs, as confirmed via TGA. The outcomes of this study suggest that the integration of CNTs and other carbon nanostructures into Ni-based and Ni-W-based hydrocracking catalysts offers new opportunities for enhancing their catalytic activity, stability, and coking resistance.

## Figures and Tables

**Table 2:** Elemental compositions and binding energies (BE) of CNT/Ni-ZY5, GNP/Ni-ZY5, CNT/Ni-ZY30, CNT/Ni-W-ZY30, GNP/Ni-W-ZY30, and their corresponding reference catalysts Ni-ZY5, Ni-ZY30, and Ni-W-ZY30, as determined via XPS.

Catalyst	Element	C 1s	O 1s	Si 2p	Al 2p	Ni 2p	W 4f	Si/Al (Atomic Ratio)	Si/Al (Nominal Ratio)
Reference catalysts									
Ni-ZY5	Atomic %	2.3	62.7	18.2	14.7	1.1	-	1.2	2.6
	BE (eV)	285.0	532.0	102.7	74.7	857.1			
Ni-ZY30	Atomic %	2.8	65.3	28.8	1.4	1.3	-	20.6	15.0
	BE (eV)	285.0	532.9	103.6	74.9	855.9			
Ni-W-ZY30	Atomic %	2.3	65.6	29.9	1.5	0.2	0.2	19.9	15.0
	BE (eV)	285.0	532.8	103.4	75.0	858.0	36.3		
CNT- and GNP-based catalysts									
CNT/Ni-ZY5	Atomic %	2.5	62.5	16.2	15.4	2.3	-	1.1	2.6
	BE (eV)	285.0	532.0	102.7	74.6	856.6			
GNP/Ni-ZY5	Atomic %	10.2	56.8	15.3	14.4	2.4	-	1.1	2.6
	BE (eV)	284.0	532.7	103.5	75.4	858.1			
CNT/Ni-ZY30	Atomic %	5.8	63.5	27.9	1.1	1.3	-	25.4	15.0
	BE (eV)	284.7	533.6	104.3	75.6	856.8			
CNT/Ni-W-ZY30	Atomic %	2.5	64.8	30.2	1.8	0.3	0.2	16.8	15.0
	BE (eV)	285.0	532.9	103.4	75.0	857.8	36.9		
GNP/Ni-W-ZY30	Atomic %	11.3	59.3	27.0	1.4	0.3	0.3	19.3	15.0
	BE (eV)	284.1	533.6	104.1	75.7	858.6	37.5		

## Data Availability

The data presented in this study are available on request from the corresponding author.

## Supplementary Materials

The following supporting information can be downloaded at: https://www.mdpi.com/article/10.3390/nano12183246/s1, Table S1: Textural properties of the CNT/Ni-ZY30 catalysts prepared under varied conditions, Table S2: EDX-TEM elemental composition of Ni-W-ZY30, Figure S1: (a) N2 adsorption–desorption isotherms at 77 K and (b) DFT pore size distributions (PSD) for the CNT/Ni-ZY30 catalysts prepared under varied conditions, Figure S2: XRD patterns of the CNT/Ni-ZY30 catalysts prepared under varied conditions, Figure S3: (a) Conversion and selectivity percentages of the main products for the CNT/Ni-ZY30 catalysts prepared under varied conditions, as well as (b-d) conversion and selectivity results over 20 h time-on-stream, Figure S4: Conversion and selectivity percentages of the main products for (a) Ni-W-ZY30, (b) CNT/Ni-W-ZY30, and (c) GNP/Ni-W-ZY30 catalysts after regenerating them for three cycles.
