# Supplementary material for: Carbon Nanostructure/Zeolite Y Composites as Supports for Monometallic and Bimetallic Hydrocracking Catalysts
Source: Nanomaterials (Basel). 2022 Sep 19;12(18):3246. doi: 10.3390/nano12183246 (PMC9504968; doi:10.3390/nano12183246)
Supplement: Supplementary file 1 [file nanomaterials-12-03246-s001.zip › nanomaterials-1893381-SI.pdf]

Supporting Material

Carbon Nanostructure/Zeolite Y Composites as Supports for Monometallic and Bimetallic Hydrocracking Catalysts

Roba Saab [1], Kyriaki Polychronopoulou [1,2,*], Dalaver H. Anjum [2,3], Nikolaos Charisiou [4], Maria A. Goula [4], Steven J. Hinder [5], Mark A. Baker [5] and Andreas Schiffer [1,*]

[1] Department of Mechanical Engineering, Khalifa University of Science and Technology, Abu Dhabi 127788, United Arab Emirates

[2] Center for Catalysis and Separations, Khalifa University of Science and Technology, Abu Dhabi 127788, United Arab Emirates

[3] Department of Physics, Khalifa University of Science and Technology, Abu Dhabi 127788, United Arab Emirates

[4] Department of Chemical Engineering, University of Western Macedonia, Koila, 50100 Kozani, Greece

[5] The Surface Analysis Laboratory, Faculty of Engineering and Physical Sciences, University of Surrey, Guild-ford GU2 4DL, UK

[*] Corresponding authors:
Email: andreas.schiffer@ku.ac.ae (A. Schiffer), kyriaki.polychrono@ku.ac.ae (K. Polychronopoulou)

S1. Catalyst textural characterization

Table S1: Textural properties of the CNT/Ni-ZY30 catalysts prepared under varied conditions.

Catalyst composition	BET surface area (m^2/g)	Pore volume (cm^3/g)		
		Micropore	Mesopore	Total
CNT/Ni-ZY30 (500C, 2h)	612	0.22	0.22	0.44
CNT/Ni-ZY30 (500C, 4h)	584	0.21	0.22	0.43
CNT/Ni-ZY30 (650C, 2h)	601	0.23	0.22	0.45
CNT/Ni-ZY30 (650C, 4h)	576	0.22	0.22	0.44

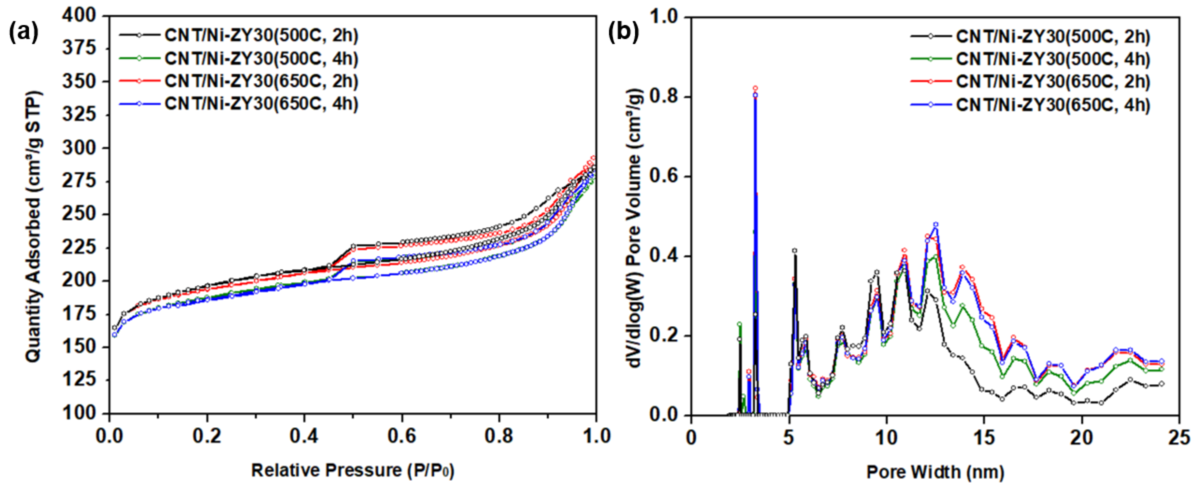

Figure S1: (a) N_2 adsorption-desorption isotherms at 77 K, and (b) DFT pore size distributions (PSD) for the CNT/Ni-ZY30 catalysts prepared under varied conditions.

S2. Catalyst structural characterization

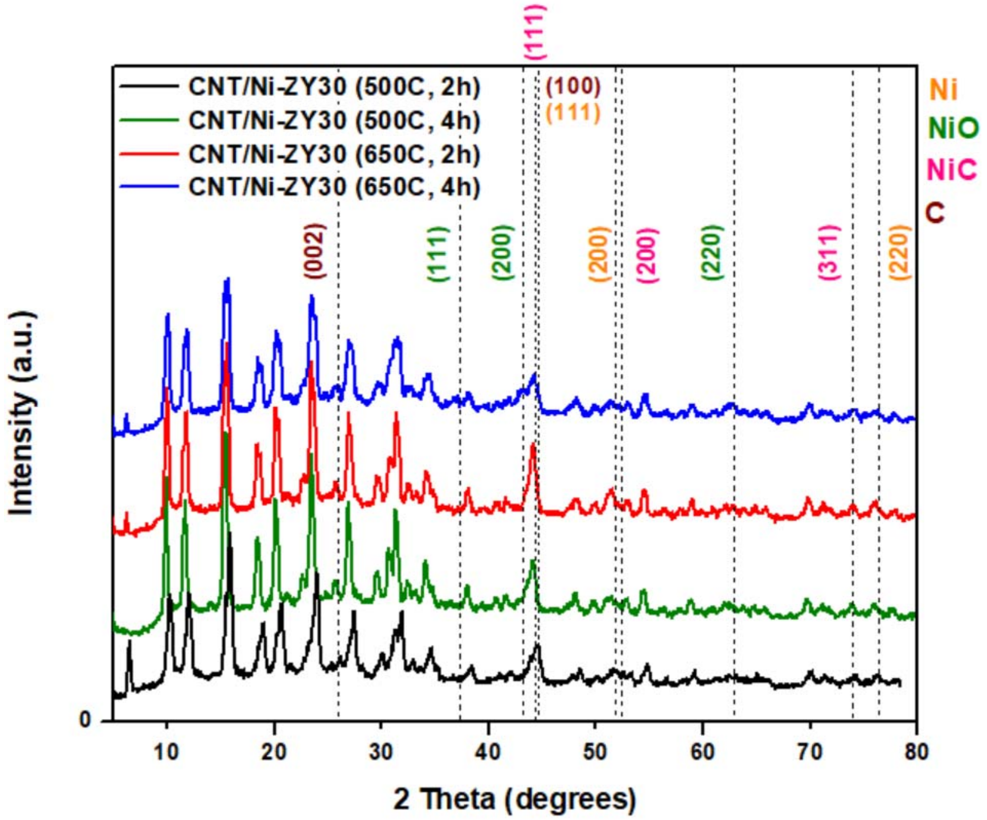

Figure S2: XRD patterns of the CNT/Ni-ZY30 catalysts prepared under varied conditions.

Table S2: EDX-TEM elemental composition of Ni-W-ZY30.

	O K	Al K	Si K	Ni K	W L	Si/Al	nominal Si/Al (atomic ratio)
Weight %	56.1	2.2	34.8	1.7	5.2	15.7	15.8
Atomic %	71.8	1.7	25.4	0.6	0.6	15.1	15.0

S3. Catalytic performance studies

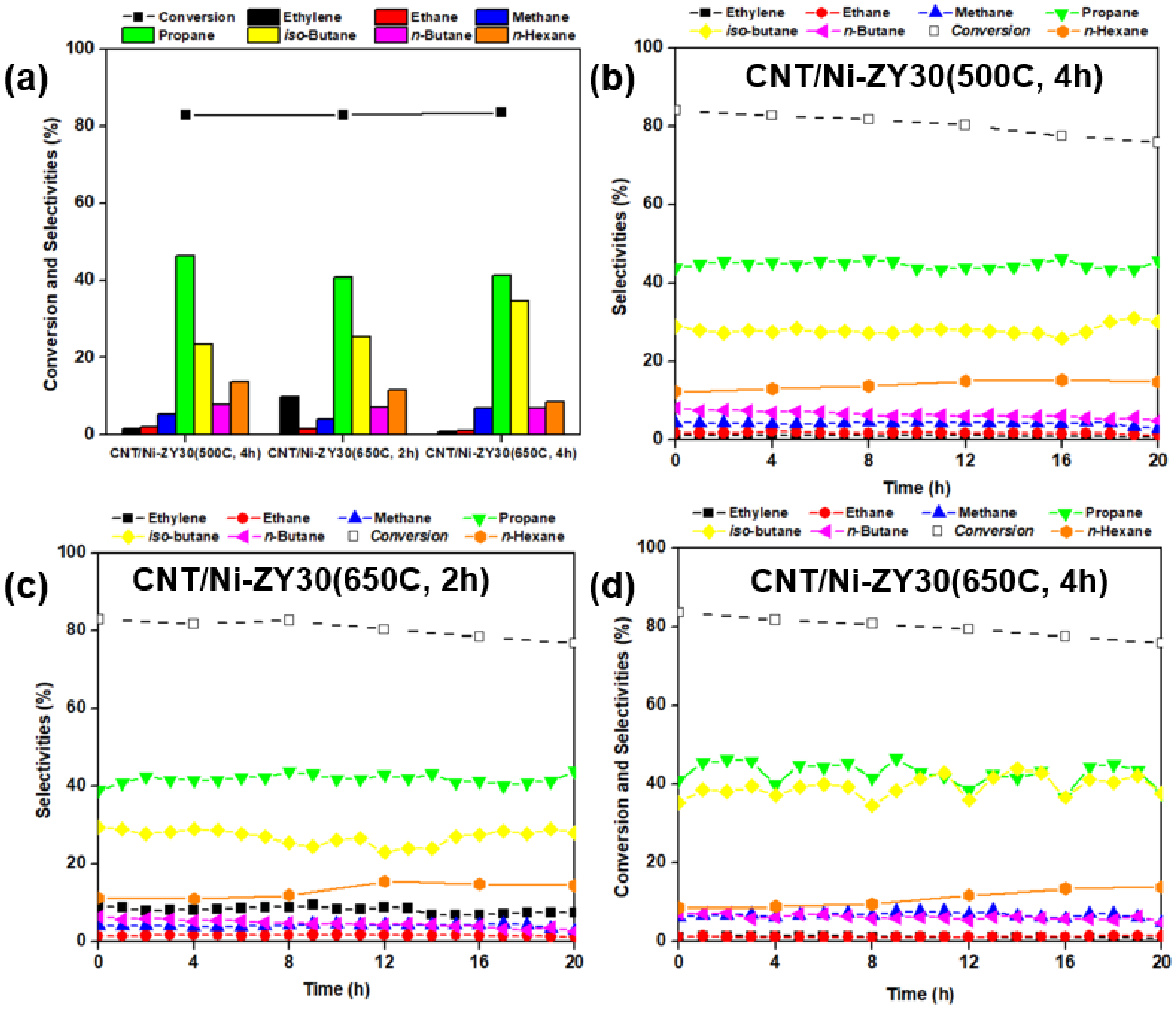

Figure S3: (a) Conversion and selectivity percentages of the main products for the CNT/Ni-ZY30 catalysts prepared under varied conditions, as well as (b-d) conversion and selectivity results over 20 h time-on-stream.

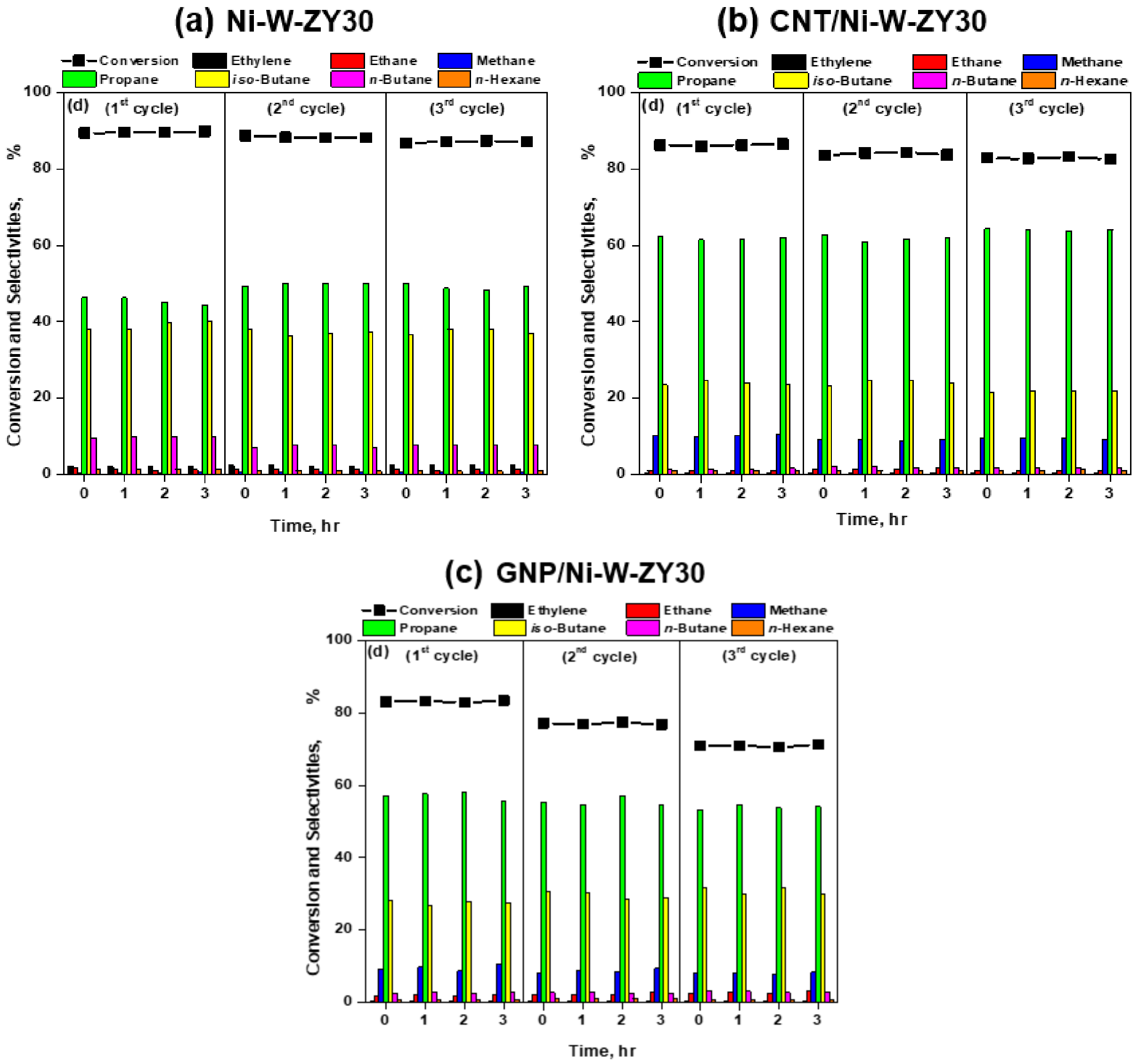

Figure S4: Conversion and selectivity percentages of the main products for (a) Ni-W-ZY30, (b) CNT/Ni-W-ZY30, and (c) GNP/Ni-W-ZY30 catalysts after regenerating them for three cycles.
